# Airy beam self-focusing in a photorefractive medium

**DOI:** 10.1038/srep35078

**Published:** 2016-10-12

**Authors:** Noémi Wiersma, Nicolas Marsal, Marc Sciamanna, Delphine Wolfersberger

**Affiliations:** 1 LMOPS, CentraleSupélec, Université de Lorraine, 57070 METZ, France.; 2 LMOPS, CentraleSupélec, Université Paris-Saclay, 57070 METZ, France.

## Abstract

The unique bending and shape-preserving properties of optical Airy beams offer a large range of applications in for example beam routing, optical waveguiding, particle manipulation and plasmonics. In these applications and others, the Airy beam may experience nonlinear light-matter interactions which in turn modify the Airy beam properties and propagation. A well-known example is light self-focusing that leads to the formation of spatial soliton. Here, we unveil experimentally the self-focusing properties of a 1D-Airy beam in a photorefractive crystal under focusing conditions. The transient evolution involves both self-bending and acceleration of the initially launched Airy beam due to the onset of an off-shooting soliton and the resulting nonlocal refractive index perturbation. Both the transient and stationary self-focusing properties can be tuned by varying the bias electric field, the injected Airy beam power and the background illumination.

Although being a truncated solution of the ideal Airy waveform, the optical Airy beam keeps its accelerating, non-spreading and self-healing properties over a finite distance^1^. The propagation of Airy beams in nonlinear media has been first studied in an unbiased photorefractive medium, where an optical beam is mainly subject to the diffusion effect. It has been shown that the peculiar asymmetrical Airy field distribution enables the Airy beam to undergo self-trapping, i.e. the diffraction of an Airy beam can be annihilated via the carrier diffusion effect in a nonlinear medium over a longer distance^2^,^3^. This observation also holds for Airy pulses^4^.

Besides the shape-preserving propagation of an Airy beam in diffusive media, the propagation direction of the accelerating beam can also be altered via an externally applied electric field. By studying further the impact of the nonlinearity of the medium on the propagation of an Airy beam, various theoretical as well as experimental studies have demonstrated that the shape and trajectory and therefore the acceleration of the Airy beam can be engineered via a refractive index variation^5^,^6^,^7^. The control of the Airy beam’s ballistic properties using either the medium nonlinearity or photonic lattices^8^,^9^ offers new possibilities in all-optical waveguiding or routing.

All these previous studies considered the nonlinear propagation of an Airy beam where the beam preserves its multi-lobe distribution and its curved trajectory (i.e. self-trapping). When applying a small bias electric field to the nonlinear medium, the Airy beam still undergoes self-trapping and the main lobe is narrower while still accelerating^3^. However, recent works have suggested the possibility to induce spatial solitons through self-focusing (and not self-trapping) of optical Airy beams^3^,^10^,^11^,^12^,^13^. When applying a larger bias electric field to the nonlinear medium, the Airy beam does not entirely turn into a soliton, but decomposes itself into a so-called off-shooting soliton and an accelerating wave packet. However these self-focusing properties of an optical Airy beam have not been experimentally demonstrated.

In this Letter we experimentally demonstrate the existence of solitonic beam structures induced by an Airy beam under strong nonlinear self-focusing conditions. When applying a bias electric field in the direction of the c-axis of a photorefractive medium, the Airy beam splits into a weak accelerating structure and an off-shooting soliton propagating along the crystal without transverse acceleration. These results match the theoretical predictions in literature^3^,^10^,^11^,^12^,^13^. By studying experimentally for the first time the transient self-focusing of the Airy beam, we unveil a two-steps dynamics. When applying an external bias voltage on the medium, the Airy beam first self-focuses into a solitonic structure as theoretically predicted. This solitonic beam coexists with an accelerating wave and, for longer times, relaxes into an Airy-like accelerating beam. By analyzing the properties of this off-shooting soliton build-up, we show that the onset of an off-shooting soliton shed from the initial Airy beam involves both self-bending and acceleration of the initially launched Airy beam. In addition, we demonstrate that both the transient and stationary self-focusing properties can be tuned by varying the bias electric field, the injected Airy beam power and an external background illumination applied on top of the photo refractive crystal [Fig. 1].

## Results

**Figure 1:**
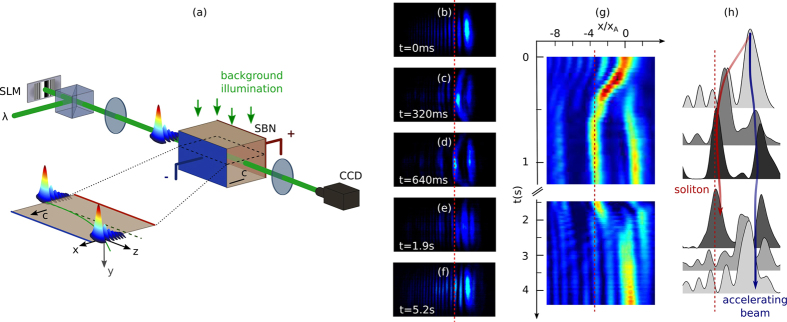
(**a**) Experimental setup. (**b–f**) Transverse intensity profile of the output beam under nonlinear focusing conditions (*E*_*e*_ = 4 kV/cm, *P*_*A*_ = 400 *μ*W) for increasing times. (**g**) 1D intensity profile along time. (**h**) Top-view sketch of the accelerating beam interacting with the off-shooting soliton, superimposed with their intensity profiles along time.

Our experiment consists of propagating a one-dimensional Airy beam (*λ* = 532 nm) into a photorefractive SBN-crystal with dimensions 5 mm*5 mm*1 cm (*n*_*SBN*_ = 2.3) as depicted on Fig. 1. The one-dimensional Airy beam is generated using a cubic phase modulation on a spatial light modulator and is defined by the initial conditions:

*ψ*(*x*)_*z*=0,*t*=0_ = *Ai*((*x* + *x*_*A*_)/*x*_*A*_)*exp*(*a*(*x* + *x*_*A*_)/*x*_*A*_), where *x*_*A*_ = 10 *μ*m is the main lobe’s waist and *a* = 0.04 the truncation parameter of the Airy beam. Under linear conditions, the beam propagates along the z-axis of the crystal with a transverse parabolic acceleration along the c-axis (parallel to the x-axis) of the photorefractive crystal. In our experiment we set *x* = 0 as the transverse output position of the linear Airy beam. 
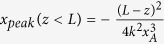
 (where *k* = 2*πn*/*λ* is the wave vector) mathematically describes the transverse parabolic acceleration. As a consequence, the Airy main lobe has initially been launched at −*x*_*peak*_(*z* = 0) = 34 *μ*m.

When an external electrical bias field *E*_*e*_ is applied along the x-axis at *t* = 0 s, the optical Airy beam photoinduces a refractive index variation in the crystal through the Pockels effect. The photorefractive effect in the SBN-crystal induces both a focusing and a shift of the optical energy along the transverse *c*-axis thanks to mainly two contributions: the drift effect induced by the electrical bias field and the thermal diffusion effect^14^. To optimize the nonlinear photorefractive and solitonic effect of our system, the external electrical bias field is set at *E*_*e*_ = 4 kV/cm. In this nonlinear regime, the propagating 1D Airy beam turns into a so-called “off-shooting soliton” along the tangential direction (*z*-axis) and an accelerating beam^13^,^15^. The theoretical output position of the off-shooting soliton then matches with the input position of the main lobe of the Airy beam, i.e. no transverse shift along the x-axis. To analyze the dynamics of this nonlinear interaction, we image the output face of the crystal through a CCD camera. Figure 1(b–f) show the evolution of the intensity profile of the output beam versus time. Starting at *t* = 0 s, the intensity shifts towards the position of the linear second lobe, further towards the higher lobe’s orders [Fig. 1(b,c)] and finally reaches a maximum transverse shift *x* = −34 *μ*m at *t* = 640 ms [Fig. 1(d)]. We will further refer to this position as the off-shooting soliton’s position (red dashed line). Then, on a longer time-scale a relaxation-type dynamics is observed towards a redistributed Airy-like profile similar to the input beam at *t* = 0 s [Fig. 1(e,f)]. The spatiotemporal dynamics of the nonlinearly propagating Airy beam can therefore be summarized in three stages. (i) First the output beam focuses towards the red dashed line of Fig. 1(g,h). (ii) Then we observe two co-existing beam’s structures [Fig. 1(g,h)]: the so-called off-shooting soliton at *x*/*x*_*A*_ = −3.7 and an accelerating structure at *x*/*x*_*A*_ = 0.5 with similar intensities. (iii) Finally the two previous solutions merge and form a new Airy-like structure on a longer time scale. Similar to the relaxation dynamics of a spatial soliton formed by a self-focused Gaussian beam^14^,^16^, the accelerating beam therefore relaxes for longer times into a less focused multi-lobe beam with a peak intensity that shifts back towards the +*x*-axis.

As we will now detail, the nonlinear interactions that emerge from the transient behavior of a single self-focused Airy beam can be characterized as an attraction of the accelerating structure towards the solitonic beam. Simultaneously the accelerating beam presents a tightening effect of the lobes. We show that both attraction and tightening effects can be tuned via the optical power of the initial Airy beam and a background illumination.

**Figure 2:**
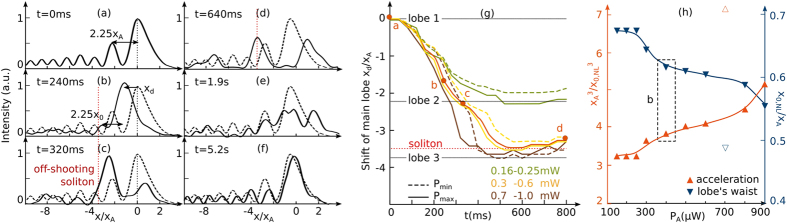
Experimental transient self-focusing of an Airy beam. (**a–f**) Intensity profile of the output beam along time, *P*_*A*_ = 400 *μ*W. (**a**) Linear intensity profile, (**b–d**) build-up of the off-shooting soliton, (**e,f**) relaxation into a multi-lobe stationary solution. The dashed lines correspond to the linear profile. (**g**) Attraction effect: transverse position of the output intensity peak versus time for different input powers. (**h**) Tightening effect: normalized acceleration and main lobe’s waist *x*_0_ of the Airy beam nonlinearly attracted towards its off-shooting soliton for increasing input power *P*_*A*_.

In order to characterize the attraction and deflection, we plot in Fig. 2 the nonlinear transverse intensity profile of the output beam for increasing times compared to the linear case at *t* = 0 s [Fig. 2(a)]. *x*_*d*_ corresponds to the shift of the accelerating wave packet induced by the attraction of the off-shooting soliton with respect to the initial launched Airy beam. The position of the initial Airy main lobe defines the zero attraction position. Thus *x*_*d*_ < 0 illustrates the attraction of the Airy beam towards the off-shooting soliton’s position (*x*/*x*_*A*_ = −3.7, see Fig. 2(c,d)). It is worth mentioning that on Fig. 2(c–e) the output profile of the accelerating structure does not match with an Airy distribution anymore, but the output beam still presents secondary lobes. This is due to the multi-channel waveguiding structure photoinduced by the multi-lobe structure of the Airy beam at *t* = 0 s. After *t* = 1.9 s, the solitonic structure vanishes and the intensity redistributes into an Airy-like profile [Fig. 2(f)]. Figure 2(g) details the temporal evolution of the transverse intensity peak’s position at the output of the crystal during the transient build-up regime of the off-shooting soliton (*t* < 800 ms, stages (i)–(ii)). Initially the Airy-distributed energy is mainly concentrated in the first lobe at *x* = 0. After the electrical switch-on of the focusing nonlinearity of the system at *t* = 0 s, the position corresponding to the peak intensity shifts towards the position of the higher lobes’ orders along the −*x*-axis (bending effect). Then, around *t* = 500 ms, the position of the peak intensity reaches a quasi-steady position corresponding to the location of the off-shooting soliton until, as depicted on Fig. 1(g), for longer times beyond t = 1 s, the position of the peak intensity shifts back into the position of the main lobe of the accelerating beam.

In a second step we now analyze whether the interactions between the solitonic and accelerating waves can be tuned by varying the nonlinearity. In what follows we vary the intensity of the input Airy beam to tune the refractive index modulation depth and analyze the corresponding attraction and tightening effects. Figure 2(g) shows the evolution of the deflection of the accelerating beam for increasing Airy beam powers. Similarly to the self-focusing properties of Gaussian beams^17^,^18^, the transient time towards self-focusing is smaller when the input light intensity increases. In addition the increase of input power modifies the transient self-focusing properties. As depicted on Fig. 2(g), when the power increases from *P*_*A*_ = 250 *μ*W to *P*_*A*_ = 300 *μ*W, the maximum shift does not increase linearly, but jumps from the former second lobe’s position (*x*/*x*_*A*_ = −2.25) to the theoretical output position of the off-shooting soliton. When further increasing the power (*P*_*A*_ > 700 *μ*W), the maximal bending of the beam still deviates but saturates at the third lobe’s position (*x*/*x*_*A*_ = −3.7). By varying the optical power it is therefore possible to balance between diffraction and nonlinearity and tune our nonlinear system from a weak interaction (*P* ≤ 250 *μ*W) to a strong attraction (*P* > 250 *μ*W) between the accelerating wave packet and the solitonic structure.

In addition, during the transient self-focusing regime we also observe a tightening of the lobes of the accelerating beam. Figure 2(h) depicts the normalized peak tightening rate *x*_0_/*x*_*A*_ of the lobes of the accelerating beam for increasing power, where *x*_0_ is computed by fitting the intensity profile with an Airy beam profile [Fig. 2(a,b)]. As the peak acceleration of an Airy beam is linked to the resulting lobes’ size *x*_0_ 
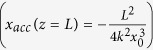
, the normalized peak acceleration for increasing input powers *P*_*A*_ is calculated as follows: (∂^2^*x*_*peak*_/∂*z*^2^)_*NL*_/(∂^2^*x*_*peak*_/∂*z*^2^)_*lin*_ = (*x*_*A*_/*x*_0,*NL*_)^3^. The resulting evolution is also displayed on Fig. 2(h). As shown by the data on Fig. 2(h), the tightening of the interlobes’ distance induces an increase of the acceleration rate from three (*P*_*A*_ = 200 *μ*W) to five times (*P*_*A*_ = 900 *μ*W) the initial value.

In summary, the self-bending of the accelerating beam towards the off-shooting soliton and its tightening effect are increased with the nonlinearity of the system during the transient self-focusing regime of an Airy beam. In particular the optical power enables to tune the effects of the self-focusing strength on the accelerating structure, hence offering an easy-to-use control parameter for interactions between the solitonic beam and the accelerating beam.

**Figure 3:**
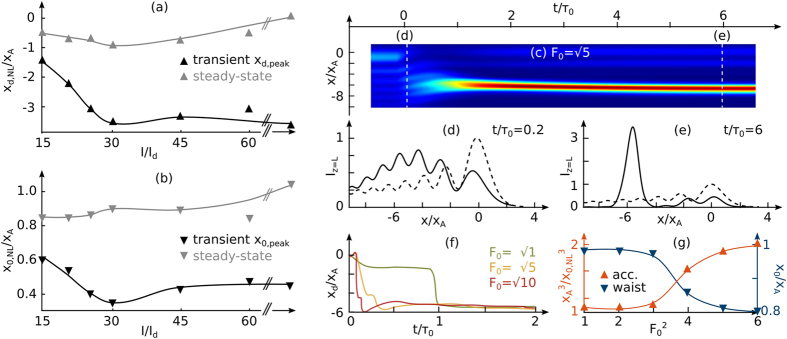
(**a,b**) Influence of background illumination on the transient and final self-focusing beam: (**a**) for the transverse beam’s shift *x*_*d*_ (attraction parameter) (**b**) and the interlobes’ distance *x*_0_ (cubic proportional to the acceleration). (**c–g**) Numerical study: Self-focusing of an Airy beam. (**c**) Intensity profile of the output beam along time; transverse intensity profiles in the (**d**) transient and (**e**) steady-state regime. The dashed lines in (**d,e**) correspond to the linear profile (*t* < 0 s). (**f**) Transverse position of the output intensity peak versus time for increasing optical power (via *F*_0_). (**g**) Acceleration effect: normalized acceleration and main lobe’s waist *x*_0_ of the Airy beam for increasing input intensity.

As already mentioned, the photorefractive nonlinearity of our system can be tuned by different physical parameters such as the external electric bias field (*E*_*e*_), the intensity of the launched beam (with 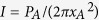 in the main lobe) but also the so-called dark intensity of the photorefractive crystal via an external background illumination [Fig. 1]. Such an illumination tends to artificially increase the dark conductivity of the photorefractive crystal which is initially very weak (*I*/*I*_*d*_ ≈ ∞). In photorefractive systems using Gaussian beams it has been shown that *I*_*d*_ plays a significant role during the self-focusing and solitonic regime^17^. In our Airy beam system, we question how such a background illumination may influence the previous results. Figure 3(a,b) depict the influence of *I*/*I*_*d*_ on the transient and corresponding steady state (*t* > 8 s) peak values of *x*_*d*_ and *x*_0_. On Fig. 3(a) for *I*/*I*_*d*_ = 15, the self-bending of the Airy beam observed previously is reduced in the transient regime from 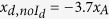 to 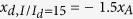. Contrary to the case without background illumination, where the Airy-like structure of stage (iii) is superimposed with the initial Airy beam [Fig. 2(f)], adding *I*_*d*_ enables the accelerating beam to remain shifted even in the steady-state regime (maximum shift of −*x*_*A*_ for *I*/*I*_*d*_ = 30). As depicted on Fig. 3(b), the background illumination also influences the self-focusing effect both in the transient and the steady-state regime. In particular we still observe self-focusing of the accelerating beam after *t* > 8 s for *I*/*I*_*d*_ = 45.

Our experimental results can be reproduced qualitatively well by numerical simulations. See Fig. 3(c–g). The nonlinear propagation of the Airy beam in the photorefractive crystal can be simulated by the normalized non-linear Schrödinger Eq. 1 suggested by Belić *et al.*^19^, where an optical beam *F*(*x*, *t*) propagates following the nonlinear wave propagation equation in a photorefractive SBN-crystal:


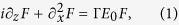



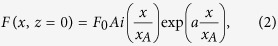



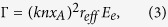


where Γ = 10 is the nonlinear photorefractive coupling strength, *r*_*eff*_ = 235*pm*/*V* is the effective component of the electro-optic tensor, *E*_*e*_ the external electric field and *E*_0_ = *E*_*sc*_/*E*_*e*_ is the homogeneous part of the *x*-component of the photorefractive space charge field. As the optical intensity modulates the space charge field, the steady-state *E*_0_ is equal to *E*_0_ = −*I*_0_/(1 + *I*_0_), *I*_0_ = |*F*|^2^. The time-dependency of the space charge field *E*_0_ is calculated from:


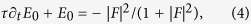


where *τ* = *τ*_0_/(1 + *I*_0_) is the relaxation time of the crystal, with *τ*_0_ the characteristic response time of the crystal. The numerical system is completely dimension-free, in particular the propagation *z*-axis is normalized to the diffraction length 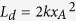 and the transverse *x*-axis is normalized to the beam’s waist being the lobe’s waist *x*_*A*_ = 10 *μ*m in the case of the Airy beam. We fix the truncation parameter *a* = 0.1 and vary the optical field amplitude *F*_0_.

Similarly to the experiment, the Airy beam undergoes self-focusing into an off-shooting soliton (at *x*/*x*_*A*_ = −5.5) and an accelerating beam (at *x*/*x*_*A*_ = 0) [Fig. 3(c–e)]. The self-bending and the acceleration effects induced by the off-shooting soliton are also observed and can be enhanced by increasing the optical power and therefore the refractive index change [Fig. 3(f,g)]. The numerical results are in good qualitative agreement with the experimental observations of the nonlinear interactions between the off-shooting soliton and the accelerating beam (stages (i) and (ii)). We note that the numerical simulations do not reproduce the relaxation-type dynamics of the beam (stage (iii)). Although the main focus here is the nonlinear interaction between the accelerating beam and the off-shooting soliton well described by the model Eqs 1, 2, 3, 4, this suggests that a more complete description of the relaxation process in self-focusing requires to account for other transport mechanisms as for example carrier diffusion.

## Conclusion and Discussion

In summary, this work is the first experimental analysis of the self-focusing properties of an Airy beam in a photorefractive nonlinear medium. The transient self-focusing behavior shows nonlinear interactions between a soliton and an accelerating beam. These interactions result in both (i) an attraction and deflection effect into the off-shooting soliton’s position and (ii) a tightening of the interlobes distance which induces an increase of the acceleration rate. Both attraction and tightening effects can be tuned via the Airy beam intensity or the background illumination.

Very recently, the nonlinear interaction between an accelerating (Airy) beam and a spatial soliton - created by the self-focusing of a Gaussian beam through thermal nonlinearity^20^ - was analyzed in the context of optical gravitational lensing. Relying on the analogy with the Newton-Schrödinger model for quantum gravity, the authors relate the long-range interactions between the spatial soliton and the accelerating beam to the effects caused by a mass on light propagating in a gravitational field. In our configuration, where a soliton and an accelerating beam co-exist during the transient self-focusing regime of an Airy beam, the off-shooting soliton shed from the initial Airy beam similarly plays the role of a mass that attracts and deflects the remaining accelerating light from its own curved trajectory. The properties of this analogous gravitational lensing, i.e. deflection and acceleration, can be both controlled all-optically through the engineering of the optical photorefractive nonlinearity. Our observations being very similar to those of ref. 20 but in a different system, our conclusion is therefore that the analogy with gravitational lensing effects is not limited to the Newton-Schrödinger framework but applies more generally to a nonlinear Schrödinger equation that accounts for a non-local nonlinearity. Besides its interest for the analogy with gravitation, the two-stages build-up dynamics of the focused beam provides a deeper insight into the subject of accelerating beams in nonlinear focusing media, and can be used to photoinduce multiple waveguide structures, as suggested in ref. 15.

## Additional Information

**How to cite this article**: Wiersma, N. *et al.* Airy beam self-focusing in a photorefractive medium. *Sci. Rep.* **6**, 35078; doi: 10.1038/srep35078 (2016).
